# Dr. E. P. Griswold

**Published:** 1918-06

**Authors:** 


**Dr. E. P. Griswold** of Niagara Falls died April 1st, age 63. He graduated from the New York University in 1878.

